# Sepsis caused by *Listeria monocytogenes* during chemotherapy for small cell carcinoma of the thymus

**DOI:** 10.1186/s13104-015-1230-9

**Published:** 2015-06-26

**Authors:** Masamichi Itoga, Yuko Asari, Takeshi Morimoto, Kageaki Taima, Kunihiko Nakamura, Yoshihito Tanaka, Hisashi Tanaka, Shingo Takanashi, Hiroyuki Kayaba, Ken Okumura

**Affiliations:** Department of Cardiology, Respiratory Medicine and Nephrology, Hirosaki University Graduate School of Medicine, 5 Zaifu-cho, Hirosaki, 036-8562 Japan; Department of Clinical Laboratory Medicine, Hirosaki University Graduate School of Medicine, 5 Zaifu-cho, Hirosaki, 036-8562 Japan; Department of Cardiology Medicine, Mutsu General Hospital, 1-2-8 Ogawa-cho, Mutsu, 035-8601 Japan; Department of Anatomic Pathology, Hirosaki University Graduate School of Medicine, 5 Zaifu-cho, Hirosaki, 036-8562 Japan; Health Administration Center, Hirosaki University, 1 Bunkyo, Hirosaki, 036-8562 Japan

**Keywords:** Bacterial translocation, Chemotherapy, *Listeria monocytogenes*

## Abstract

**Background:**

*Listeria monocytogenes* is a facultative intracellular parasitic bacterium that is Gram positive, catalase positive, oxidase negative, and a facultative anaerobe. It is known to infect humans through food. It is a bacillus with low virulence, but can cause meningitis and sepsis in infants and immunocompromised patients.

**Case presentation:**

A case of 75-year-old Japanese female with small cell carcinoma of the thymus and pleural dissemination is described. She was treated with carboplatin and etoposide and showed a partial response. However, the tumor recurred 6 months later. Therefore, we again administered carboplatin and etoposide. Though peritoneal dissemination was suspected based on abdominal computed tomography findings after two courses, the assessment was stable disease. She was occasionally treated for constipation. She developed chills, rigor, and diarrhea, necessitating admission on the 7th day of the third course of chemotherapy. We suspected intestinal infection, and cefepime was thus administered. However, her blood pressure dropped and neutropenia manifested on the 4th day of admission. We therefore switched the antibiotic from cefepime to meropenem and also administered granulocyte-colony stimulating factor. *Listeria monocytogenes* was detected by two blood cultures, and the antimicrobial medication was thus switched to ampicillin, in consideration of sensitivity. Her general condition improved and she was able to leave the hospital on the 19th day after admission.

**Conclusions:**

During chemotherapy, factors such as impaired bowel movements, malnutrition, and myeloablation can contribute to the development of severe infections. It is necessary to comprehensively assess a patient’s state and treat all aspects of illness.

## Background

*Listeria monocytogenes* (*L. monocytogenes*) is a facultative intracellular bacterium that is Gram positive, catalase positive, oxidase negative, and a facultative anaerobe. *L. monocytogenes* is known to infect humans through food including cheese and fresh vegetables [1]. It is a bacillus with low virulence, and this infection is rare in adults without underlying diseases, but it can cause meningitis and sepsis in infants and the elderly, as well as in immunocompromised patients [2]. There are some reports of Listeriosis in Japan, in the compromised host during chemotherapy [3] and after surgery [4, 5]. In a Japanese review of listeriosis cases over for 23 years, it was reported that 76.4% of deaths in adult cases had underlying conditions [6].

We experienced a case of sepsis caused by *L. monocytogenes* during chemotherapy for small cell carcinoma of the thymus. This case is reported with a discussion of the relevant literature.

## Case presentation

**Figure 1:**
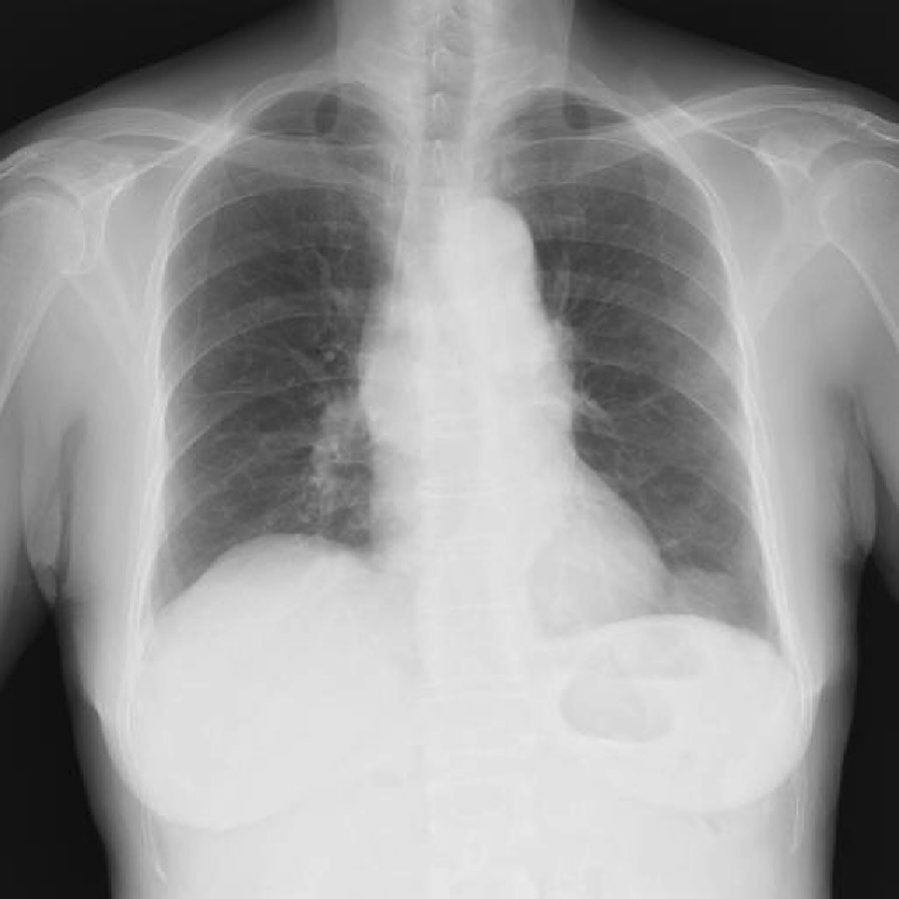
Chest X-ray showed no abnormal findings.

**Figure 2:**
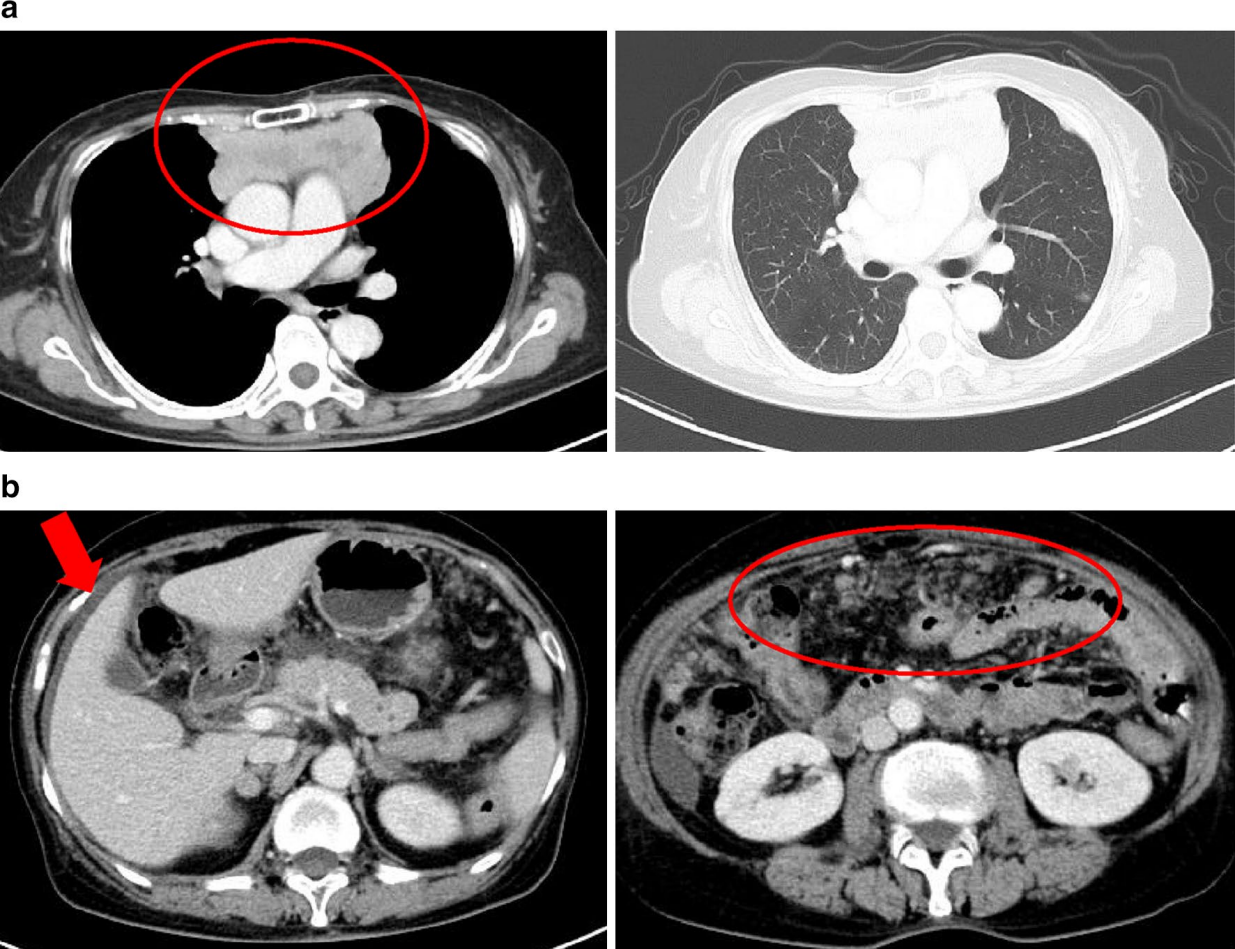
**a** Chest computed tomography showed an increase in the size of the small cell carcinoma tumor arising from the thymus (*red circle*). **b** Abdominal computed tomography showed mild ascites (*red arrow*) and prominent irregular granular shadows of the greater omentum and the mesentery (*red circle*).

The patient was a 75-year-old Japanese female who presented with chief complaints of fever and diarrhea. The past history, family history, and personal/occupational history were unremarkable. A diagnosis of small cell carcinoma of the thymus (cT4N2M1 stage IV) was made on January 19, 2010. Chemotherapy was started with carboplatin (CBDCA) + etoposide (VP-16) (Chemotherapy for small cell carcinoma of the thymus has not established. We performed the chemotherapy for small cell of the thymus using the regimen for small cell lung carcinoma as reference.) on the same day, and a total of four courses were administered. The tumor decreased in size following this treatment and the response was assessed as partial response (PR). Relapse of the tumor was detected in July, and chemotherapy with CBDCA + VP-16 was resumed due to sensitive relapse. Findings on chest-abdominal computed tomography (CT) performed for evaluation on October 12 after two courses of treatment suggested the possibility of peritoneal dissemination. However, the tumor response was assessed as stable disease (SD), and the treatment was continued. While receiving this treatment, the patient complained of constipation, which was dealt with symptomatically. On December 21 (the 7th day of the third course of chemotherapy), the patient developed chills, fever, and diarrhea, and was admitted on December 22 with a diagnosis of severe infection as an adverse event during chemotherapy. Vital signs were normal, except for a body temperature of 38.7°C. Chest auscultation revealed no abnormal findings. Examination of the abdomen also revealed no abnormalities, except for slight generalized tenderness. Laboratory examination revealed the following: hematology: pancytopenia (WBC 2,900/μL, RBC 335 × 10^4^/μL, Hb 10.2 g/dL, Plt 10.1 × 10^4^/μL); blood biochemistry: slight deterioration of liver function and electrolyte abnormalities (AST 37 U/L, ALT 30 U/L, LDH 250 U/L, ALP 454 U/L, γ-GTP 214 U/L, Na 132 mmol/L, Cl 95 mmol/L), and significant increase in serum CRP (CRP 32.76 mg/dL); blood coagulation profile: elevation of serum levels of FDP (FDP 22.5 μg/mL); serum tumor marker levels: within normal range. Arterial blood gas analysis revealed evidence of respiratory alkalosis (pH 7.532, pCO2 28.5 Torr, pO2 70.4 Torr, HCO3 23.8 mmol/L). Although *Enterococcus faecalis* was detected in the urine, urine microscopy revealed only 5-9 WBC/HPF, not suggestive of urinary tract infection (Table 1). Influenza test was negative. A chest X-ray revealed no abnormal findings (Figure 1). Chest CT showed an increase in the size of the small cell carcinoma tumor arising from the thymus in the anterior mediastinum (Figure 2a). Abdominal CT revealed mild ascites and prominent irregular granular shadows of the greater omentum and the mesentery (Figure 2b), suggestive of peritoneal dissemination.

**Table 1:** Laboratory data at initial visitation

CBC		Blood chemistry		Tumor marker	
WBC	2900/μL	AST	37 IU/L	NSE	8.4 ng/mL
Neutro	92.5 %	ALT	30 IU/L	ProGRP	43.7 pg/mL
Mono	0.3 %	Y-GTP	214 IU/L	Urinalysis	
RBC	335 × 10^4^/μL	LDH	250 IU/L	WBC	5–9/HPF
Hb	10.2 g/dL	ALP	454 IU/L	*E. faecalis*	1 × 10^7^
Pit	10.1 × 10^4^/μL	TP	6.7 g/dL	Arterial blood gas analysis (room air)	
Coagulation		Alb	3.4 g/dL	PH	7.532
PT-INR	1.15	CK	21 IU/L	pCO_2_	28.5 Torr
APTT	33.4 s	BUN	18 mg/dL	pO_2_	70.4 Torr
Fibrinogen	352 mg/dL	Cre	0.66 mg/dL	HCO_3_	23.8 mmol/L
FDP	22.5 μg/mL	Na	132 mmol/L	SaO_2_	95.5 %
AT-III	85%	K	4.1 mmol/L	Bacteriological examination	
		CI	95 mmol/L	Blood	*L. monocytogenes* (+)
		Ca	8.3 mmol/L		
		CRP	32.76 mg/dL

The focus of the infection was unclear, but since the patient was a compromised host, enteric bacterial infection was assumed and treatment was started empirically with cefepime (CFPM) 3 g/day. Samples were collected for two sets of blood culture at the time of admission. Because of a fall in blood pressure and neutropenia on December 25, the antimicrobial drug was switched to meropenem (MEPM) 1.5 g/day, and administration of granulocyte-colony stimulating factor (G-CSF) was started. On December 26, *L. monocytogenes* was identified in both sets of blood culture bottle, in BacT/ALERT blood cultures by direct inoculation into the Vitek 2 system (This serotype is not known.). On December 28, with recovery of the neutrophil count, the antibiotic was again changed to ampicillin (ABPC) 4 g/day in consideration of the bacterial sensitivity. The patient then recovered gradually and was discharged on January 10, 2011.

## Discussion

*L. monocytogenes* is widely distributed throughout the environment including soil, water, and decaying vegetation. *L. monocytogenes* exists in the feces of animals and putrescent tissues, showing a high osmotic pressure tolerance and the ability to proliferate in temperatures ranging from 1 to 45°C [2]. The gastrointestinal mucous membrane is considered to be the most common source of infection [1].

*L. monocytogenes* has the ability to pass the intestinal barrier. The primary method of entry into endothelial cells is believed to be via a zipper-like mechanism [7]. Invasion proteins on the surface of the bacteria, like Internalin A, Internalin B, and P60, help the bacterium bind to host surface receptors [8]. This ability is thought to trigger bacterial translocation (BT).

BT is considered to be the mechanism underlying sepsis due to *L. monocytogenes*. The phenomenon of enteral bacteria moving outside of the intestinal tract was described a century ago in animal experiments [9]. BT was defined as “the passage of viable bacteria from the gastrointestinal tract through the epithelial mucosa into the lamina propria and then to the mesenteric lymph nodes and possibly other organs” [10]. The intestinal tract then came to be thought of as a new source of infection. At present, microbial translocation, i.e., the concept of BT, is recognized as involving not only live bacteria but also components of dead bacteria and bacterial factors such as endotoxin [11]. *L. monocytogenes* is thought to invade intestinal epithelial cells and the mucous membrane lamina propria via various routes and to become an internalization of macrophages and lymphocytes, subsequently migrating to remote organs through lymphatic vessels [7].

The mechanisms of BT are considered to be (1) a change in enterobacterial flora, (2) failure of the intestinal tract barrier, and (3) diminished host immunity. The enterobacterial flora maintains an equilibrium allowing fungi and bacteria to coexist. When this enterobacterial flora balance is compromised by antimicrobials, antacids, ileus, or fecal impaction, pathogenic microbes and toxins proliferate abnormally in the intestinal tract, and this leads to contact with intestinal epithelia. The intestinal tract barrier is an epithelial structure that functions as a mechanical barrier and also provides a mucous membrane layer to protect the organism from excessive intestinal tract peristalsis and digestive juices such as gastric acid and bile acid. With the atrophy of intestinal tract disuse, ischemia may occur due to shock, malnutrition, hepatic cirrhosis, arterial clotting, inflammatory bowel disease, or radiation exposure, leading to barrier function failure, and pathogenic microbes can then come into contact with the intestinal mucosa. The intestinal mucosa then becomes hyper-permeable, facilitating invasion by pathogenic microbes. The intestinal tract has an immunologic barrier function and harbors local immune factors including neutrophils, macrophages, and gut-associated lymphoid tissue (GALT) of the terminal ileum. GALT is the most immunologically active tissue in the human body. Decreasing immune cells in GALT, due to nutritional management designed to avoid the gastrointestinal tract, reduces local immunity by causing IgA secretion disorders in the intestinal epithelia, compromised host immunity, and diminished host immuno-responsiveness to invasive pathogens from the intestinal tract. These alterations can all promote invasion by pathogens. It is thought that myeloablation with chemotherapy and the associated reduction in host immunity, direct damage to the intestinal mucosa by chemotherapy, intestinal tract obstruction by tumors with peritoneal dissemination, and constipation due to impaired bowel movements associated with disease states or treatment can lead to BT.

Normalization of the aforementioned processes is necessary for the prevention and treatment of BT. In other words, for prevention of BT it is necessary to (1) control the enterobacterial flora, (2) maintain the intestinal tract barrier, (3) control immuno-responsiveness, and (4) maintain the intestinal tract blood supply. To achieve the prevention and treatment of BT, attention is being paid to the administration of probiotics and prebiotics [12, 13], glutamine [14], polyethylene glycol [15], and dobutamine [16]. In addition, to control immuno-responsiveness, endotoxin adsorption with blood adsorption therapy using a polymyxin B column was reported to be useful [17, 18].

As in our case, with the source of infection being unclear, the sepsis often progresses. BT is potentially involved in the infectious process by various mechanisms and must, in the intestinal tract, be regarded as an important source of infection. Thus, it is necessary to prevent BT as well as to implement infection control procedures.

## Conclusion

We encountered a case with sepsis caused by *L. monocytogenes* during chemotherapy for small cell carcinoma of the thymus. We often experience unidentified sepsis during chemotherapy. Impaired bowel movements, malnutrition, and myeloablation by chemotherapy can easily cause bacterial translocation. We must not forget the possibility of serious sepsis caused by *L. monocytogenes* through bacterial translocation during chemotherapy. It is necessary to comprehensively assess a patient’s state and treat all aspects of illness.

### Consent

Written informed consent was obtained from the patient for publication of this Case Report and any accompanying images. A copy of the written consent is available for review by Editor-in-Chief of this journal.

## Abbreviations

ABPC: ampicillin
BT: bacterial translocation
CBDCA: carboplatin
CFPM: cefepime
CT: computed tomography
GALT: gut-associated lymphoid tissue
G-CSF: granulocyte-colony stimulating factor
*L. monocytogenes*: *Listeria monocytogenes*
MEPM: meropenem
PR: partial response
SD: stable disease
VP-16: etoposide
